# Reduced inflammatory and muscle damage biomarkers following oral supplementation with bioavailable curcumin

**DOI:** 10.1016/j.bbacli.2016.02.003

**Published:** 2016-02-18

**Authors:** Brian K. McFarlin, Adam S. Venable, Andrea L. Henning, Jill N. Best Sampson, Kathryn Pennel, Jakob L. Vingren, David W. Hill

**Affiliations:** a Applied Physiology Laboratory, University of North Texas, Denton, TX, United States; b Department of Biological Sciences, University of North Texas, Denton, TX, United States

**Keywords:** Inflammation, Cytokines, Multiplex, Leg press

## Abstract

**Background:**

Exercise-Induced Muscle Damage (EIMD) and delayed onset muscle soreness (DOMS) impact subsequent training sessions and activities of daily living (ADL) even in active individuals. In sedentary or diseased individuals, EIMD and DOMS may be even more pronounced and present even in the absence of structured exercise.

**Methods:**

The purpose of this study was to determine the effects of oral curcumin supplementation (Longvida® 400 mg/days) on muscle & ADL soreness, creatine kinase (CK), and inflammatory cytokines (TNF-α, IL-6, IL-8, IL-10) following EMID (eccentric-only dual-leg press exercise). Subjects (N = 28) were randomly assigned to either curcumin (400 mg/day) or placebo (rice flour) and supplemented 2 days before to 4 days after EMID. Blood samples were collected prior to (PRE), and 1, 2, 3, and 4 days after EIMD to measure CK and inflammatory cytokines. Data were analyzed by ANOVA with P < 0.05.

**Results:**

Curcumin supplementation resulted in significantly smaller increases in CK (− 48%), TNF-α (− 25%), and IL-8 (− 21%) following EIMD compared to placebo. We observed no significant differences in IL-6, IL-10, or quadriceps muscle soreness between conditions for this sample size.

**Conclusions:**

Collectively, the findings demonstrated that consumption of curcumin reduced biological inflammation, but not quadriceps muscle soreness, during recovery after EIMD. The observed improvements in biological inflammation may translate to faster recovery and improved functional capacity during subsequent exercise sessions.

**General significance:**

These findings support the use of oral curcumin supplementation to reduce the symptoms of EIMD. The next logical step is to evaluate further the efficacy of an inflammatory clinical disease model.

## Highlights

• Oral optimized curcumin supplementation reduced biological indices of muscle inflammation following exercise-induced muscle damage.
• Oral optimized curcumin supplementation did not significantly reduced subjective quadriceps muscle soreness for this sample size following exercise induced muscle damage.
• These findings support the use of oral, optimized curcumin supplementation to reduce the biological symptoms associated with exercise induced muscle damage.
• The next logical step is to evaluated oral, optimized curcumin supplementation in an inflammatory clinical disease model.

## 1 Introduction

Resistance exercise is a key component of a comprehensive exercise training program for both athletes and recreationally active individuals. Although resistance exercise is an effective means to increase lean muscle mass in the long-term, in the short-term, resultant exercise induced muscle damage (EIMD) and soreness can limit performance in subsequent training sessions or competitive events [10]. Also EIMD models are relevant because they can be used to mimic what happens with various clinical conditions. Generally, delayed onset muscle soreness (DOMS) and inflammation peak between 1 and 2 days following a resistance exercise session. Non-steroidal anti-inflammatory drugs (NSAIDs) are commonly used to treat DOMS [17]. While the reduction of some inflammation may be therapeutic, the severe blunting of inflammation caused by NSAID blocks or impairs the initial stage of healing. Despite the perceived benefits of NSAID, clinical research demonstrates that NSAID use does not necessarily improve recovery following EIMD [20]. Moreover, in some users, regular NSAID use has been reported to exert negative effects on the central nervous system [1]. Thus, investigating less damaging, naturally occurring, substances that have anti-inflammatory actions is warranted.

While many different substances have been studied as a treatment for EIMD and DOMS, curcumin is of special interest because it is purported to act through a similar mechanism as NSAID, albeit with a less pronounced suppression of inflammation [24]. As such, curcumin acts by modifying COX-2 pathway signaling, which results in reduced inflammatory cytokine (i.e. IL-1β, IL-6, IL-8, and/or TNF-α) and prostaglandin production [9], [13]. This modulation could be important since prostaglandins play a role in increasing the severity of subjective pain following EIMD and IL-8/TNF-α have emerged as stable blood biomarkers of inflammation [2], [18], [22]. The known ability of curcumin to reduce in vitro COX-2 signaling makes it an ideal candidate to treat EIMD and DOMS; however, only a handful of studies have examined this effect [6], [7], [12], [14], [19], [21], [23]. Of these published studies, two have used murine models that do not translate well to humans [6], [12] and the human studies either did not use a pure eccentric EIMD model (i.e. they used downhill running or strenuous endurance exercise) [7], [14], [19], [21], [23] or they used an impractical dose of naturally occurring curcumin [19]. Another potential limitation with oral curcumin supplementation is that not all curcumin supplements deliver free curcumin into the blood [9]. Those that don't deliver free curcumin, deliver curcumin metabolites that have a short half-life, are not readily absorbed by target tissues, and have low bioactivity [9]. Given the limited number of previous publications there is clearly a need to complete additional research to characterize the potential of curcumin supplementation to treat EIMD and DOMS.

Another important factor to consider in the evaluation of previous studies involving curcumin is the form of curcumin used. In its naturally occurring state, curcumin has very low bioavailability in humans [3] and, therefore, doses from murine models do not scale to reasonable doses in humans [6], [12]. For example, when considering a study conducted by Davis et al. [6], a comparable dose in humans would need to include at least 5 g/day of oral curcumin ingestion (~ 25, 400 mg capsules), which is not practical. With, a 5 g dose of naturally occurring curcumin, the amount of bioactive curcumin present in the blood would likely be less than 200 mg/day (< 5% bioavailability of naturally occurring curcumin) [9]. Recently, various forms of curcumin that are “optimized” to address low bioavailability issues have become commercially available [9].

The present study sought to expand the existing literature by designing a study that first identified an optimal dose of commercially available, “optimized” curcumin needed to elicit a specific biological effect (i.e. reduced serum TNF-α). We did not focus on measurements of COX-2 or prostaglandins because the relationship of these outcomes to curcumin supplementation is established. This optimal dose was then used to evaluate the efficacy of curcumin as a treatment for muscle soreness and biological inflammation following EIMD. Beyond implications for EIMD, the findings of the present study have implications for clinical conditions where chronic muscle damage occurs. The purpose of this study was to determine the effects of oral curcumin supplementation (400 mg/day) on subjective quadriceps muscle soreness, serum creatine kinase (CK), and serum inflammatory cytokines following 60 repetitions of eccentric-only dual-leg press exercise at 110% of the 1RM.

## 2 Materials and methods

### 2.1 Ethical approval

The Institutional Review Board (IRB) of the University of North Texas approved all procedures described in this report. All subjects provided their verbal and written consent prior to participating in the study. All procedures were completed according to the standards set by the latest revision of the *Declaration of Helsinki*.

### 2.2 Design and determination of dosing

Prior to performing the present study, we completed a pilot experiment (N = 20 subjects) to compare the effect of three oral doses of curcumin (Longvida®; 200, 400, and 1000 mg) against a placebo to identify an optimal dose needed to lower serum inflammatory cytokines (data not shown). A pilot dose experiment was warranted because doses used in murine model studies do not translate well to human models and, to our knowledge, no existing human studies have provided consistent information identifying an optimal dose to alter serum inflammatory cytokines following EIMD. In this pilot study, we found that a curcumin dose of 400 mg/day resulted in at least a 19% blunting of serum TNF-α at either 1 or 2 days following EIMD compared to placebo (effect size = 0.42). TNF-α blunting with the 200 mg/day dose was substantially less (6%) with a very small effect size (0.20). The 1000 mg/day dose did not result in a significantly great blunting of TNF-α than the 400 mg/day dose (minimum detection was 17%; effect size = 0.44 compared to placebo). Therefore, a dose of 400 mg/day was selected for the current study design. This study was powered based on changes in serum TNF-α, which in our pilot experiment had the smallest effect size and has been reported to change consistently following EIMD [11]. By powering the study on the ability of curcumin to blunt changes in serum TNF-α, it was calculated that a minimum of 12 subjects per group was needed (total N = 40). In order to account for anticipated subject attrition/non-compliance (determined to be 45% in our pilot experiment), we enrolled 20 subjects per group. Subject attrition/non-compliance was broadly defined to include subjects that did not adhere to the study protocol (i.e. missed supplement doses, missed study appointments, did not report to the lab fasted, etc.), those that we could not collect a blood sample on due to dehydration or vascular anatomy, and subjects who asked to drop. In the case of the present study, no subjects asked to drop, thus the key source of attrition was associated with lack of adherence to the study protocol or sample collection.

### 2.3 Study population

Interested subjects were recruited from the campus by announcements and word of mouth. Exclusion criteria included: regular resistance training in the previous 6 months, leg muscle or orthopedic condition, arthritis or other chronic inflammatory injury in the lower extremity, regular ingestion (> 2 times per week) of curcumin containing foods, regular NSAID use (at least 3 of 7 days), intake of a curcumin supplement (within the past 6 months), and any other condition that would prohibit completion of the lower body resistance exercise protocol. We experienced 30% attrition/non-compliance (as defined above) in the current study and 28 subjects completed the entire protocol (Table 1).

### 2.4 Muscle strength testing and familiarization

Approximately 10 days prior to the muscle damage session, subjects were scheduled to report to the laboratory to complete a muscle strength test. After being provided 5 min of dynamic warm-up on a cycle ergometer, a light resistance (based on gender, age, and body weight) was loaded onto a 45° inclined seated dual leg press and subjects were familiarized with the exercise movement. Targeted cadence for the repetitions (4 s concentric and 5 s eccentric) was maintained using a metronome. After demonstration, the resistance was increased and subjects were asked to complete as many repetitions as possible using the prescribed cadence. The number of repetitions was recorded and the 1 repetition maximum (1RM) was estimated using a standard method [15]. The subject was scheduled to report back to the laboratory 2 days after the initial testing session to repeat the strength test. The second 1RM estimate was used for the calculation of workloads for the muscle damage protocol. The interclass coefficient between the 1st and 2nd 1RM strength tests was > 95%.

### 2.5 Supplement conditions

Stratified randomization (based on gender and initial strength) was used to assign subjects to the treatment conditions to ensure similar numbers of men and women and a balance based on initial muscle strength between conditions. Commercially available curcumin supplements were used for the present study (Longvida®; Verdure Sciences Corp.; Noblesville, IN). Subjects were provided supplements using a double-blind approach beginning 2 days prior to EIMD and continuing until 3 days after EIMD. Subjects consumed either 400 mg of rice flour (placebo) or curcumin daily (total study dose = 2400 mg) depending on the condition they were assigned to. Capsules were ingested after rising in the morning and following an overnight fast (> 8 h). Subjects reported no adverse gastrointestinal effects. With the exception of the 2 days prior to EIMD, subjects took their recovery supplements (PRE, 1, 2, 3, and 4 dayspost-EIMD) in the presence of a member of the study staff. During the 2 days prior to EIMD, subjects confirmed supplementation ingestion via a text message to the study staff. Subject compliance during this phase was 100% for the subjects who completed all study requirements. Subjects who failed to take the supplement as prescribed were dismissed from the study and their samples were not analyzed.

### 2.6 Exercise-Induced Muscle Damage (EIMD) protocol

Subjects completed 6 sets of 10 repetitions of the leg press exercise with a beginning load set at 110% of their estimated 1RM. As in the familiarization sessions, subjects were asked to maintain a 5-s eccentric contraction on the leg press, with the assistance of a metronome. The 5-s eccentric phase was selected to maximize the duration of eccentric contraction and thus EIMD [25]. Two spotters assisted the subjects during the concentric phase of contraction to reduce muscle fatigue. During subsequent sets, if a subject was unable to maintain the 5-s eccentric contraction duration the resistance was reduced by 2.2 kg for that set and subsequent sets. Every precaution was taken to carefully adjust leg press resistance so that the subject could maintain the prescribed speed of eccentric contraction. Sets were separated by 5 min of passive, seated rest.

### 2.7 Subjective quadriceps muscle soreness

The dual leg press exercise involved several different lower extremity muscle groups, and we made our measures of soreness on the anterior thigh (i.e. quadriceps) and used those as representative of the other muscles in the leg. Subjective quadriceps muscle soreness was measured at three anatomical locations (distal, middle, and proximal) using an existing method [16], [25]. Briefly, a standard force (consistent within subject, 20–30 N) was applied using a force gauge over the targeted anatomical site in the seated position with the knee fully extended and relaxed. Subjects were provided a 10 cm line with no visual markers other than “anchors” at each end and asked to indicate their level of soreness by crossing the line at a point they felt corresponded to their soreness. In order to determine soreness score, the same researcher “scored” each rating scale using a ruler by measuring the distance from the left side “anchor” to the location the subject “crossed.” The three ratings for each quadriceps were summated to determine total quadriceps soreness scores for the right and left legs.

### 2.8 Activities of daily living (ADL) soreness

In addition to measuring subjective, palpated quadriceps soreness, we also assessed the subject's soreness during completion of common activities of daily living (ADL). To accomplish this, we used a modified version of the Knee injury and Osteoarthritis Outcome Score (KOOS) designed to ask about muscle and joint soreness and its impact of activities of daily living during the previous 1-day period. Values expressed are arbitrary units with higher scores representing greater levels of soreness while completing ADLs.

### 2.9 Blood sample collection

Venous blood samples were collected prior to (PRE), 1, 2, 3, and 4 dayspost-muscle damage using standard phlebotomy technique from an antecubital vein. Blood was collected into a serum separator tube and allowed to clot. Once clotted, the blood was centrifuged (1500 ×* g* for 20 min). Isolated serum was either analyzed for creatine kinase concentration or frozen at − 80 °C until cytokine analysis.

#### 2.9.1 Measurement of serum creatine kinase

Creatine kinase (CK) was measured in triplicate using a commercially available enzymatic assay (Pointe Scientific; Canton, MI) on an automated chemistry analyzer (ChemWell-T; Awareness Technologies; Palm City, FL). Interassay CV was < 8%.

#### 2.9.2 Measurement of serum cytokines

Serum concentrations of IL-6, IL-8, IL-10, and TNF-α were measured according to our previously described multiplex technique [17]. Briefly, aliquots of serum were analyzed in duplicate using a high-sensitivity bead-based multiplex assay, according to the manufacturer's recommendations (Milliplex; EMD Millipore; St. Louis, MO). Acquired raw data files were used to determine unknown concentrations using Milliplex Analyst Software (EMD Millipore). Interassay CV was < 9%.

### 2.10 Statistical analysis

Prior to formal statistical testing, data were checked for normality and constant error variance. Data found to be in violation of these assumptions were normalized using a log-transformation. Data were statistically analyzed for significance using a 2 (Group: curcumin or placebo) by 5 (time: PRE, 1, 2, 3, and 4 dayspost-muscle damage) analysis of variance (ANOVA) with repeated measures on the second factor (time). Significance was set to P < 0.05 and significant alpha values were adjusted using the Huynh–Feldt method to account for the repeated measures design. Location of significance effects was determined using individual *t*-tests with a Bonferroni correction for multiple comparisons. All data are reported as the mean ± SE unless otherwise indicated.

### 2.11 Heat map multivariate data visualization

One challenge associated with working with multivariate data sets is that it can be difficult to visualize global changes with all outcome variables. This potential point of confusion was addressed by generating heat map plots to visualize all biomarker changes as a function of condition and measurement time (Fig. 4). We used a color scheme where dark green represented the smallest response and dark red represented the largest response within a given outcome variable. Any response that fell between small and large was shaded an intermediate color ranging between green and red (see scale bar in Fig. 4). Responses were color coded across outcome variable (i.e. CK, TNF-α, etc.) regardless of condition (i.e. placebo vs. curcumin).

## 3 Results

### 3.1 Subjective quadriceps muscle soreness

**Fig. 1:**
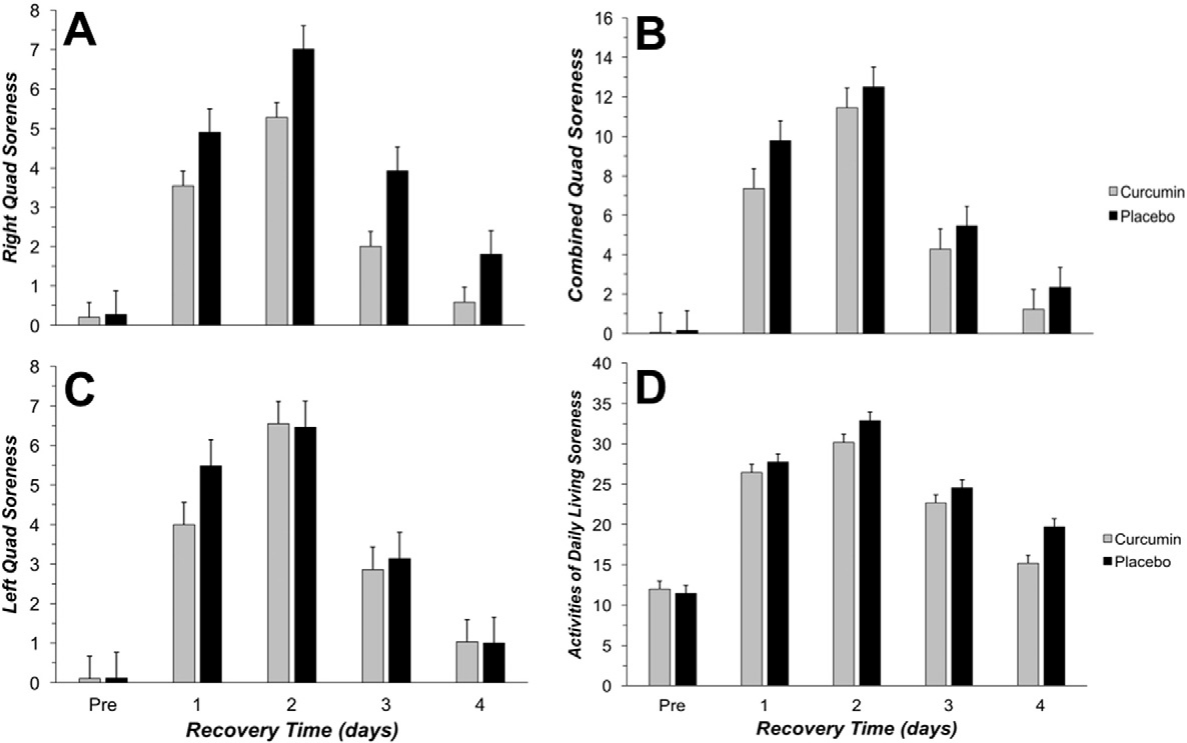
Subjective quadriceps muscle and ADL soreness was consistently used to assess perceived muscle soreness using a visual analog scale. Measurements were made prior to (PRE), 1, 2, 3, and 4 days following EIMD (Exercise-Induced Muscle Damage; 60 reps at 110% of the 1RM, eccentric only). Black bars represent placebo and gray bars represent curcumin. Summated right (A), left (B), and combined (C) quadriceps soreness was assessed. Also ADL soreness (D) was assessed using a standard scale. † indicated leg with a significant (P < 0.05) increase from PRE across both groups at 1, 2, and 3 days following EMID. There was no significant difference in muscle soreness between treatment conditions.

There was a significant main effect for time associated with subjective muscle soreness for left quad (Fig. 1A; P = 0.001), right quad (Fig. 1B; P = 0.002), and combined quad (Fig. 1C; P = 010). Peak muscle soreness was observed at 2 days after EIMD regardless of how the soreness was combined. There was no significant difference in muscle soreness between supplement conditions over time.

### 3.2 Activities of daily living soreness

There was a significant main effect for time associated with ADL soreness (Fig. 1D; P = 0.25). Peak ADL soreness scores were reached at 2 days after EIMD and mirrored the changes observed in subjective quadriceps muscle soreness. There was no significant difference in ADL soreness between supplement conditions over time.

### 3.3 Serum creatine kinase

**Fig. 2:**
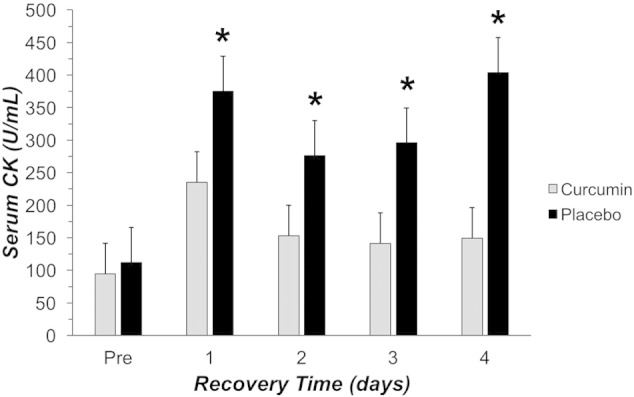
Serum creatine kinase (CK) measured prior to (PRE), 1, 2, 3, and 4 days following EIMD (Exercise-Induced Muscle Damage; 60 reps at 110% of the 1RM, eccentric only). Black bars represent placebo and gray bars represent curcumin. * indicated a difference between curcumin and placebo groups (P < 0.05).

There was a significant interaction (Fig. 2; P = 0.035) where curcumin resulted in a blunted CK response at 1 day (− 44%), 2 days (− 49%), 3 days (− 57%), and 4 days (− 69%) following EIMD compared to placebo. Both conditions experienced a significant increase in CK following EIMD; however, the curcumin group had a significantly smaller, blunted increase in CK that returned to baseline values at 2 dayspost-damage. The placebo group on the other hand had a much larger 1 day peak in CK and a second peak that occurred at 4 dayspost-damage.

### 3.4 Serum cytokines

**Fig. 3:**
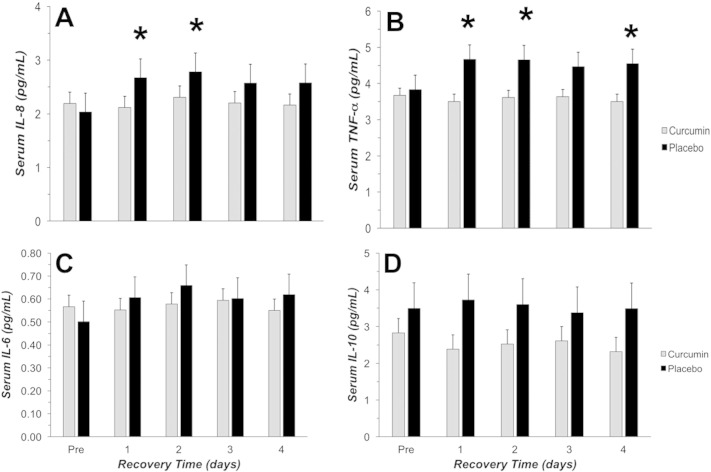
Serum cytokines (IL-8 (A), TNF-α (B), IL-6 (C), and IL-10 (D)) measured prior to (PRE), 1, 2, 3, and 4 days following EIMD (Exercise-Induced Muscle Damage; 60 reps at 110% of the 1RM, eccentric only). Black bars represent placebo and gray bars represent curcumin. * indicated a significant difference between curcumin and placebo groups (P < 0.05).

Significant interaction effects for curcumin supplementation were found for both IL-8 (Fig. 3A; P = 0.030) and TNF-α (Fig. 3B; P = 0.028); however, changes in IL-6 (Fig. 3C) and IL-10 (Fig. 3D) did not reach statistical significance for this sample size. IL-8 was significantly lower with curcumin at 1 day (− 21%) and 2 days (− 18%) compared to placebo. TNF-α was significantly lower with curcumin at 1 day (− 25%), 2 days (− 23%), and 4 days (− 23%) compared to placebo. TNF-α also trended toward being lower with curcumin at 3 days (− 19%). Despite the lack of significance for IL-6, it did appear to demonstrate a response similar to that of IL-8 and TNF-α (Fig. 3).

## 4 Discussion

The key finding was that oral curcumin supplementation (400 mg/day) significantly blunted serum creatine kinase, IL-8, and TNF-α concentrations during recovery from EIMD. Despite reduced biological indices of inflammation, we found no significant reduction in subjective quadriceps muscle soreness or ADL soreness with curcumin compared to placebo for the current sample size. This finding was somewhat expected because we powered our study to detect changes in TNF-α, but not necessarily changes in muscle soreness. Given the nature of subjective muscle soreness measures and perceived soreness during ADL, these measures tend to have a lower effect size than more stable biological measures. Our findings agree in some ways (reduced CK and inflammatory cytokines), but disagree in other ways (no reduction in muscle soreness) with previously published reports involving curcumin and EIMD [6], [7], [19], [21], [23]. Based on what is known about oral curcumin supplementation in humans, it is plausible that this may be the primary source of variability to response between studies. While the use of curcumin to modulate recovery from EIMD is still an emerging field of study, the studies that have used naturally occurring curcumin in humans report either none or few biological effects, despite very high doses used [19], [21]. Nicol et al. [19] used a murine study [6] to select a human dose of 5000 mg/day of naturally occurring curcumin, which likely resulted in a dose of < 5% of bioavailable curcumin (< 200 mg/day of bioactive curcumin) being administered [9]. Consequently, Nicol et al. [19] reported no alterations in biological indices of EIMD and only improvements in subjective muscle soreness. In our pilot dosing experiment (data not shown), we determined that a bioactive dose of at least 400 mg/day was needed to alter biological indices of inflammation. Thus, the naturally occurring dose used in the study by Nicol et al. [19] likely did not supply enough free curcumin to elicit changes in inflammation biomarkers. Interestingly, they reported reduced subjective muscle soreness with curcumin. Our findings were exactly the opposite in that we observed improvements in biomarkers of inflammation, but these did not translate into reduced quadriceps muscle soreness with curcumin. Given the mechanism of action of curcumin (i.e. COX-2 signaling), it is difficult to explain how it would be possible to reduce soreness independent of changes in inflammatory cytokines. Inflammation on the other hand is regulated by a variety of pathways, so it is plausible that curcumin exerted an independent effect on inflammation because it was acting through one of those pathways.

Our key biological outcome was that curcumin supplementation reduced serum IL-8, TNF-α, and CK following EIMD. In our study curcumin supplementation appeared to cause time differences in TNF-α (suppression maintained for up to 4 days) and IL-8 (suppression maintained for up to 2 days) response. Both of these cytokines are pro-inflammatory and thus the reduction we observed was consistent with a curcumin-induced reduction in secondary chemical damage to the muscle, which is the primary cause of the biphasic DOMS response [5], [25]. Our findings with respect to pro-inflammatory cytokines are consistent with reports in mice [6], [12], albeit with a lower daily dose of curcumin. In humans, Drobnic et al. [7] reported that another commercially available form of curcumin (400 mg/day) reduced IL-8 at 2 h after downhill running. When comparing peak CK concentrations between the present study (Peak = 400 U/mL) and that of Drobnic (Peak > 750 U/mL), it is reasonable to speculate that our measured reduction in IL-8 at 1 day may correspond with the Drobnic reduction reported at 2 h. Also, downhill running induces a more rapid muscle damage curve (as measured by serum CK) due to oxidative stress created by the higher muscle oxygen consumption of running [7], [23]. Our observed CK reduction with curcumin was consistent with what others have reported using a variety of EIMD models [7], [23]. Our time course of CK response following EIMD did differ from other studies, but this can be explained by comparing differences in the quantity of muscle damage caused during the EIMD session [11]. As mentioned, the amount of damage caused in the present study was much less than that in the study by Drobnic et al. (downhill running), but more than in the study by Nicol et al. (single leg eccentric contractions).

The ability of curcumin to alter inflammation has been fairly consistently reported; however, measurement of perceived muscle soreness and ADL soreness has yielded inconsistent reports [7], [14], [19], [21], [23]. Based on previous mechanistic work [4], [13], the assumption would be that muscle soreness reduction was a result of altered prostaglandins from decreased COX-2 signaling, which should also decrease inflammatory cytokines. In addition to potential differences in signaling pathways, muscle soreness can be influenced by an individual's perception [8]. In contrast, decreased inflammatory cytokines is a physiological process with no apparent psychological component in the context of EIMD. The findings from other investigators that curcumin reduced muscle soreness independent of reduced inflammation support the hypotheses that either different signaling pathways are involved or there is an unaccounted for psychological component related to curcumin ingestion [19], [21]. It should be noted that in the literature there are dozens of accepted ways to measure muscle soreness. In the current study we used an investigator-initiated measure of muscle soreness; however, it is possible that had we used a different technique, we may have found a different response. Method differences aside, it is also plausible that differences reported with respect to reduced muscle soreness may be attributed to placebo effect associated with curcumin supplementation. From a practical perspective, muscle soreness does not necessarily impact function. In contrast, muscle inflammation directly interferes with muscle function (i.e. reduced range of motion, reduced contractile ability, etc.). Thus, between these two outcomes, a curcumin-induced reduction in inflammation represents a greater potential to allow for more rapid muscle recovery.

**Fig. 4:**
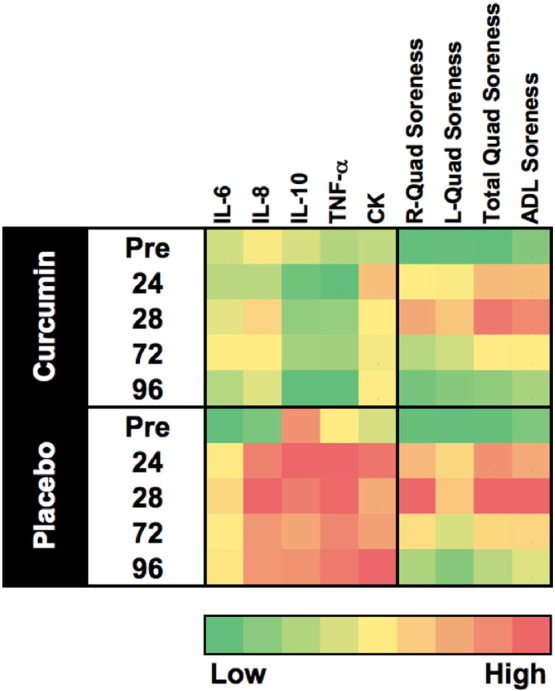
Heat map for global changes. A standard heat map was generated using a green (smallest response) to red (largest response) color scale to graphically represent each biomarker change (i.e. CK, TNF-α, etc.) as a function of treatment condition (curcumin vs. placebo). The included scale demonstrates the full range of responses. Measurements were made prior to (PRE), 1, 2, 3, and 4 days following EIMD (Exercise-Induced Muscle Damage; 60 reps at 110% of the 1RM, eccentric only). This figure demonstrates that curcumin treatment was associated with a muted response for serum cytokines (IL-6, IL-8, IL-10, and TNF-α) and creatine kinase (CK). Further interpretation reveals that while not significant at this sample size, the muscle soreness response for curcumin treatment matches with the inflammation biomarkers, particularly at 1 and 2 days. Globally this figure illustrates the close relationship between the biomarkers used in this study to evaluate recovery following EIMD.

One challenge of studies that involve multiple outcome variables is that it can be complicated to integrate the findings to create a profile of the observed treatment response. We addressed this challenge by using heat map plotting of key outcome variables (Fig. 4). This approach allowed us to present a curcumin response profile following EIMD that incorporated all of our key outcome variables. Based on the heat map it appears that biomarkers of muscle damage (CK) and inflammatory cytokines (IL-6, IL-8, and TNF-α) were blunted in a similar manner compared to placebo. While we did not detect significant treatment differences for quadriceps muscle or ADL soreness, there does appear to be slightly less soreness at 1 day with curcumin. Another interesting observation is that by 4 days the curcumin supplemented group had rectified muscle damage and inflammation, while these responses were still elevated in placebo. Our observed curcumin response profile (i.e. blunted CK, IL-8, and TNF-α) was consistent with what has been collectively reported in the scientific literature [6], [7], [14], [19], [21], [23].

In summary, the novel finding of the present study was that oral supplementation with “optimized” curcumin (400 mg/day) was associated with a blunting of serum CK, IL-8, and TNF-α for up to 4 days following EIMD. A major strength of the present study is that we had completed a pilot experiment to evaluate curcumin dose response, and we were able to properly power the present study to detect curcumin-induced changes in markers of inflammation. It is plausible to speculate that observed biological reductions may be due to curcumin-induced reduction in secondary chemical injury to the muscle during recovery. Unfortunately, the present study does not resolve confusion regarding the ability of curcumin to reduce muscle soreness following EIMD. While we carefully controlled all aspects of the study and the measurements made, no experimental study is without limitations; however, we believe that the strength of the findings outweighs any of our shortcomings. Future research should seek to systematically examine the nature of the relationship between oral curcumin dose, blood curcumin concentration (via LC–MS), blood inflammatory cytokines, and the EIMD/DOMS time course.

## Transparency document

Transparency document.

## Figures and Tables

**Table 1:** Subject characteristics.

Characteristic	Curcumin (n = 16)	Placebo (n = 12)
Gender (# F)	11	7
Age (y)	20 ± 1	19 ± 2
Height (M)	1.66 ± 0.09	1.70 ± 0.08
Weight (kg)	62.4 ± 11.4	65.0 ± 10.3
BMI	22.7 ± 3.5	22.3 ± 1.9
Body fat (%)	24.7 ± 9.4	23.3 ± 12.1
Muscle strength (1RM)	145 ± 42	202 ± 67
Total load lifted during EIMD (kg)	8972 ± 2670	12537 ± 4171

Values represent the mean ± SD.
